# Initial Fitness Recovery of HIV-1 Is Associated with Quasispecies Heterogeneity and Can Occur without Modifications in the Consensus Sequence

**DOI:** 10.1371/journal.pone.0010319

**Published:** 2010-04-26

**Authors:** Antonio V. Bordería, Ramon Lorenzo-Redondo, Maria Pernas, Concepción Casado, Tamara Alvaro, Esteban Domingo, Cecilio Lopez-Galindez

**Affiliations:** 1 Virologia Molecular, Centro Nacional de Microbiología (CNM), Instituto de Salud Carlos III, Majadahonda, Madrid, Spain; 2 Centro de Biología Molecular “Severo Ochoa” (CSIC-UAM), Madrid, Spain; 3 Centro de Investigación Biomédica en Red de Enfermedades Hepáticas y Digestivas (CIBERehd), Barcelona, Spain; National AIDS Research Institute, India

## Abstract

**Background:**

Fitness recovery of HIV-1 “in vitro” was studied using viral clones that had their fitness decreased as a result of plaque-to-plaque passages.

**Principal Findings:**

After ten large population passages, the viral populations showed an average increase of fitness, although with wide variations among clones. While 5 clones showed significant fitness increases, 3 clones showed increases that were only marginally significant (p<0.1), and 4 clones did not show any change. Fitness recovery was not accompanied by an increase in p24 production, but was associated with an increase in viral titer. Few mutations (an average of 2 mutations per genome) were detected in the consensus nucleotide sequence of the entire genome in all viral populations. Five of the populations did not fix any mutation, and three of them displayed marginally significant fitness increases, illustrating that fitness recovery can occur without detectable alterations of the consensus genomic sequence. The investigation of other possible viral factors associated with the initial steps of fitness recovery, showed that viral quasispecies heterogeneity increased between the initial clones and the passaged populations. A direct statistical correlation between viral heterogeneity and viral fitness was obtained.

**Conclusions:**

Thus, the initial fitness recovery of debilitated HIV-1 clones was mediated by an increase in quasispecies heterogeneity. This observation, together with the invariance of the consensus sequence despite fitness increases demonstrates the relevance of quasispecies heterogeneity in the evolution of HIV-1 in cell culture.

## Introduction

Experiments of virus evolution in cell culture have been employed to study the relevance of genetic concepts for RNA viruses (including Muller's ratchet, the Red Queen hypothesis, genetic drift in bottleneck transfers, among others; as review see [1]). Such studies have been also extremely useful in the understanding of the generation and quantification of genetic variation of human immunodeficiency virus type 1 (HIV-1) [2], [3], [4].These investigations showed that HIV-1 populations are composed of swarms of related variants, often centered around a high frequency variant or master sequence, forming what is known as viral quasispecies [5], [6]. According to the theoretical quasispecies model, proposed by Eigen [7], viral evolution does not operate at the level of the individual genome but rather the target of selection is the quasispecies as a whole [6].

Recently, this concept has been experimentally demonstrated for RNA viruses with the implication of quasispecies diversity in the pathogenesis of poliovirus [8], [9], and other RNA viruses [10]. In addition, the pathogenicity of a Mumps vaccine strain has been related to the quasispecies heterogeneity [11]. High fidelity viral strains that produce less heterogeneous and less pathogenic viral populations are under study as new poliovirus vaccine candidates [12].

The viral population size changes during natural HIV-1 infections. The transmission to a new individual often implies a reduction in the population size, known as transmission bottleneck, which affects the composition of the quasispecies in the recipient host [13]. Once the infection is successful in the recipient host, viruses replicate generating diversity and expanding their populations to explore additional regions of the sequence space. In a similar way, antiretroviral treatments,such as highly active antiretroviral therapy (HAART), dramatically reduce the viral load producing a population bottleneck that may be shaping virus evolution. In previous studies “*in vitro*” by our group, we documented a rapid HIV-1 fitness decrease after serial bottlenecks passages [3], and also rapid fitness recovery attained when the virus was subjected to large population passages [3], [4], [14], [15], [16]. Furthermore, we found that a very limited number of mutations in the consensus HIV-1 genomic sequences was responsible for the fitness changes [4].

To understand the variables that affect fitness recovery, ten HIV-1 clones (two of which were passaged in duplicate), whose capacity to produce infectious progeny had decreased by plaque-to-plaque transfers, were subjected to ten large population passages. The molecular changes associated with fitness recovery were analyzed by comparing the consensus genomic nucleotide sequence as well as the sequences of individual molecular clones. We detected fixation of mutations in the consensus sequences only in 7 out of the 12 passaged viruses. Heterogeneity analysis of the mutant spectra in the viral populations showed an increase of complexity along the passages and an overall positive correlation between quasispecies heterogeneity and fitness increase. These results provide the first evidence that in the course of HIV-1 evolution, mutant spectrum complexity can be a determinant of virus behavior.

## Results

### HIV-1 fitness increase after ten large population passages

**Figure 1:**
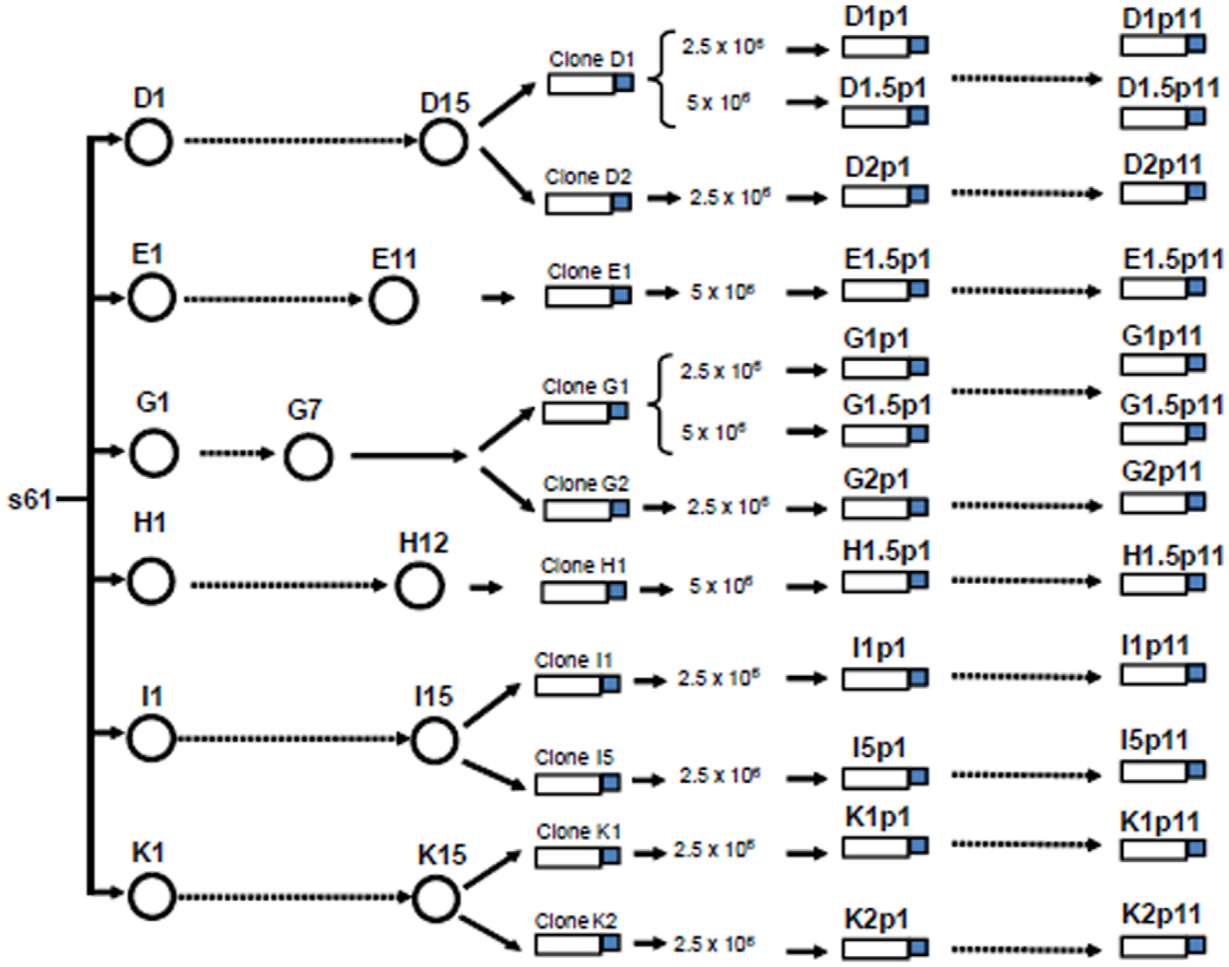
Genealogy of the viral clones studied. Representation of the serial plaque-to-plaque transfers (circles) and large population passages (bottles and dotted arrows) in 2.5×10^6^ and 5×10^6^ MT-4 cells performed with HIV-1 clones. The experimental procedures and the origins of natural isolate s61 and of the different clones are described in references [2], [3] and in Materials and methods. HIV-1 clones were obtained from the populations after the plaque-to-plaque transfers, and are named according to the number of large population recovery passages. Viral populations are indicated by letters followed by a number that indentifies the clone used, followed by p1 for the initial and p11 for the final passage. For clones D1, G1, E1 and H1, number 5 after the dot indicates that these clones were passaged by infecting 5×10^6^ MT-4 cells.

**Figure 2:**
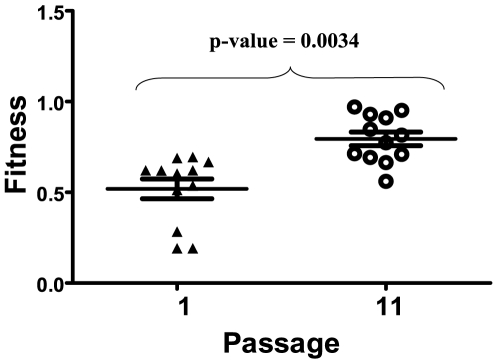
Fitness increase after ten serial passages of HIV-1 clones. Fitness values were obtained from values of each clone at passage 1 (▴) and 11 (○) and represented together with the mean value and standard errors. A Wilcoxon signed rank test was used to compare the values; the p-value of this test is shown above the points (Sum of signed ranks = −70).

Fitness of HIV-1 viral clones previously subjected to serial plaque-to-plaque transfers [3] was recovered after ten large population passages (Figure 1). Viral fitness was determined using J1 clone as reference [4]. The initial clones showed a mean viral fitness of 0.5418 (standard error (SEM)±0.054), whereas for the corresponding passaged populations, the average fitness was 0.795±0.037, a difference that was statistically significant (Wilcoxon signed rank test; p = 0.0034, Figure 2). This result indicates that few large population passages are sufficient to increase viral fitness of HIV clones that had undergone bottleneck passages. The increase in fitness, however, varied substantially among clones, with increases ranging from 386% to undetectable (0%) (Table 1). Five of the clones showed increases that were statistically significant at p<0.05; 3 clones were marginally significant (at p<0.10), and in 4 clones there was no fitness gain.

**Table 1:** Viral clones, fitness increases and mutations fixed in the consensus sequences.

Viral Clones								
Initial	Final	Fitness (SEM)	Increase(%)	p-value	Number of mutations	Localization^a^	Change of nucleotide	Change of aa
**D1p1**		0,2 (0,1)						
	**D1p11**	0,9 (0,06)	376	0.0003	1	U5 5′ LTR (593)	G à T	NA
	**D1.5p11** d	0,9 (0,07)	386	0.0013	1	U5 5′ LTR (593)	G à T	NA
**D2p1**		0,3 (0,03)						
	**D2p11**	1,0 (0,03)	243	<0,0001	1	U5 5′ LTR (593)	G à T	NA
**E1.5p1**		0,7 (0,04)						
	**E1.5p11** d	0,8 (0,03)	29	0.0017	10	U3 5′LTR (255)	G à A	NA
						U5 5′LTR (572)	T à C	NA
						*env* (2293)	G à A^b^	D249N (gp41)
						*rev (197)*		R66Q (rev)
						*rev* (209)	T à C	L70P
						*rev* (349)	T à C	STOP117Q
						*env* (2580)	T à A	Synonymous
						*nef* (426)	C à T	Synonymous
						*nef* (436)	C à G	V146L
						*nef* (544)	G à A	V182I
						*nef* (550)	A à G	R184G
**G1p1**		0,6 (0,05)						
	**G1p11**	0,8 (0,03)	25	0.0664	0			
	**G1.5p11** d	0,7 (0,02)	15	0.2329	4	*pol* (1054)	A à G	K194E (RT)
						*pol* (2346)	T à A	D64E (IN)
						*pol* (2767)	G à A	A205T (IN)
						*rev* (100)	A à G^b^	T34A (rev)
						*tat* (239)		D80G (tat)
**G2p1**		0,7 (0,03)						
	**G2p11**	0,7 (0,01)	3	0.7815	4	*pol* (1060)	G à A	E196K^c^ (RT)
						*pol* (1062)	G à A	
						*pol* (1063)	C à A	Q197K^c^ (RT)
						*pol* (1065)	G à A	
**H1.5p1**		0,5 (0,04)						
	**H1.5p11 ** d	1,0 (0,01)	85	<0,0001	3	*gag* (654)	G à A	M86I (p24)
						*env* (1549)	A à G	T1A (gp41)
						*nef* (117)	-à A	Phase recovery
**I1p1**		0,5 (0,08)						
	**I1p11**	0,7 (0,02)	29	0.0565	0	-	-	-
**I5p1**		0,6 (0,03)						
	**I5p11**	0,6 (0,03)	−7	0.6645	0	-	-	-
**K1p1**		0,7 (0,004)						
	**K1p11**	0,8 (0,03)	17	0.0963	0	-	-	-
**K2p1**		0,7 (0,04)						
	**K2p11**	0,7 (0,03)	0	0.9494	0	-	-	-
**Total**					**24**			

a Location of mutations is assigned taking HIV-1 strain HXB-2 as a reference (Accession number #K03455). Mutations are numbered according to their position in the gene. NA: not applicable. Reversions are underlined. Amino acid substitutions are numbered in reference to the protein affected.

b Nucleotide substitution producing an amino acid change in two proteins.

c Both substitutions lead to the same amino acid change.

d Viruses passaged in 5·10^6^ MT-4 cells are doubly underlined.

### Viral infectivity and p24 production in HIV-1 clones

**Figure 3:**
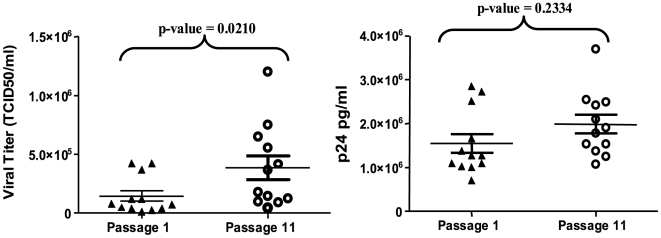
Virological characterization of HIV-1 clones in the initial and final passages. A) Viral titers of populations at passage 1 and 11 were obtained by infecting MT-2 cell with the corresponding supernatants, and are expressed as TCID_50_ (see Materials and methods). To compare the mean values, a Wilcoxon signed rank test was used (sum of signed ranks = −58) and the p-value is given above the data points. B) p24 protein was quantified using an indirect ELISA, as described in Materials and methods. Mean values of passages 1 and 11 are shown as pg/ml, and were used to compare both passages. The Wilcoxon p-value is displayed above the points (sum of signed ranks = −32). Symbols are as in Figure 2.

To identify viral factors that could be responsible for the fitness increase, viral titers and p24 protein levels were quantified for all HIV-1 clones before and after the large population passages. Passages resulted in a statistically significant increase in viral titer (Wilcoxon signed rank test; p-value = 0.021) (Figure 3a). To test whether this result was due to an increase in the virion production, viral p24 protein was quantified in the cell culture supernatants. No differences in the protein levels were observed during the passages (Wilcoxon signed rank test; p-value = 0.2334)(Figure 3b). These results suggest that the observed increase in viral titer is due to an increase of the virus infectivity, and not to an increase in virion production.

### Detection of mutations in the consensus nucleotide sequences

Because mutations are generally considered the principal cause of changes in viral fitness and other phenotypic alterations [17], [18], [19], [20], [21], [22], [23], we compared the consensus nucleotide sequence of each clone before and after being subjected to large population passages. Few mutations were fixed in each viral clone following large population passages (average of 2 mutations per genome, range 0 to 10) (Table 1). Only 7 out of the 12 populations studied showed changes in their genomic sequence, which included four reversions (17% of the changes), one mutation that eliminated a stop codon in nef coding region of virus H (Table 1) and a common mutation in the 5′LTR in three viruses, as described [4]. The results confirm, in agreement with our previous work, that few mutations in the consensus sequence can mediate fitness recovery [4].

Of the 7 clones showing accumulation of mutations, five displayed statistically significant fitness increases, whereas two clones (G1.5p11 and G2p11) did not show any significant fitness increase (Table 1). In addition, there were clones that acquired several mutations (such as clone E1) and manifested a lower fitness increase than clones that acquired a single mutation (clones in lineage D). Strikingly, clones G1p11, I1p11 and K1p11, that did not include any mutation in the consensus sequence, displayed an increase in fitness (with p = 0.0664, p = 0.0565 and p = 0.0963 respectively in the Graphpad slope comparison test). Therefore, fitness increase was not directly correlated with the number of mutations in the consensus sequence, and in some clones, this fitness increase could not be linked to any mutation in the consensus sequence of the population.

### Quasispecies heterogeneity and viral fitness

**Figure 4:**
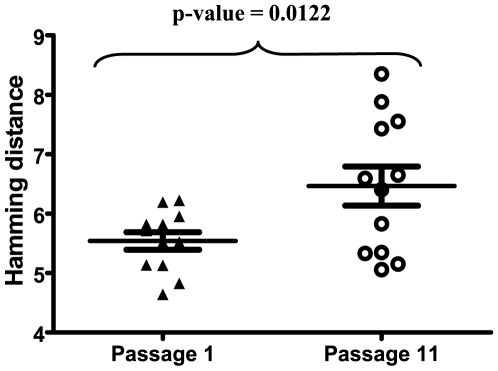
Global heterogeneity of the HIV-1 viral quasispecies at passages 1 and 11. Heterogeneity was measured as Hamming distance. Wilcoxon p-value to compare means is shown above the dots (sum of the signed ranks = −62). Symbols are as in Figure 2.

To investigate viral factors, other than dominant mutations that could be involved in fitness increase, we quantified the heterogeneity of mutant spectra, by sequencing molecular clones of four genomic regions of the initial clones and the corresponding passaged populations. At least 20 clones were analyzed for each of the four regions, which spans 2,005 nucleotides per clone, and thus represents 480,144 nucleotides. The distribution of mutations among the clones and genomic regions is described in Table 2. The analysis documented that there was a significant difference in heterogeneity (Wilcoxon signed rank test p = 0.0122), quantified as the sum of Hamming average distances in each region, between the initial (5.546±0.1481 substitutions) and the passaged viral populations (6.464±0.33 substitutions) (Figure 4).

**Table 2:** Quasispecies heterogeneity per region and clone.

	LTR-gag		vpu		env(V1–V2)		env(V3–V4)	
PASSAGE	1	11	1	11	1	11	1	11
CLONE	Mean ± SEM	Mean ± SEM	Mean ± SEM	Mean ± SEM	Mean ± SEM	Mean ± SEM	Mean ± SEM	Mean ± SEM
D1	1.0±0.3	1.1±0.4	1.2±0.4	0.6±0.2	1.9±0.5	1.5±0.4	1.7±0.4	2.1±0.4
D15	1.0±0.3	2.5±0.6	1.2±0.4	0.6±0.2	1.9±0.5	1.5±0.4	1.7±0.4	2.0±0.5
D2	2.1±0.4	3.3±0.5	0.2±0.2	1.0±0.3	0.9±0.3	1.4±0.4	2.5±0.5	2.2±0.5
E1	2.0±0.4	2.3±0.5	0.7±0.3	0.6±0.3	1.8±0.4	2.2±0.5	1.5±0.4	2.3±0.4
G1	2.1±0.5	1.7±0.5	0.2±0.2	0.5±0.2	1.2±0.4	1.5±0.4	2.0±0.4	2.2±0.5
G15	2.1±0.5	1.6±0.4	0.2±0.2	0.4±0.2	1.2±0.4	2.2±0.5	2.0±0.4	1.1±0.3
G2	1.1±0.3	2.9±0.5	0.8±0.3	0.8±0.4	1.4±0.4	2.2±0.6	1.8±0.4	2.4±0.5
H1	2.1±0.5	2.2±0.7	0.7±0.3	0.8±0.3	1.4±0.4	2.3±0.6	2.0±0.5	2.3±0.5
I1	1.8±0.4	1.2±0.4	0.1±0.1	0.2±0.2	1.2±0.4	1.6±0.5	2.0±0.5	2.0±0.5
I5	2.2±0.5	2.5±0.5	0.1±0.1	0.4±0.2	0.8±0.3	0.9±0.4	1.5±0.4	1.4±0.4
K1	1.2±0.4	1.9±0.4	0.7±0.3	0.5±0.2	1.3±0.4	2.0±0.4	1.6±0.4	2.0±0.5
K2	2.2±0.5	2.5±0.5	0.5±0.2	0.5±0.2	1.7±0.5	1.6±0.4	1.8±0.4	2.0±0.5
Mean/Region	1.7±0.1	2.1±0.2	0.6±0.1	0.6±0.1	1.4±0.1	1.7±0.1	1.8±0.1	2.0±0.1

Heterogeneity is measured using Hamming distance and the results show the mean value per clone in each region for passage 1 and 11. The final row shows the heterogeneity average value of the passages in each region. Values between regions cannot be compared as we are expressing them as number of differences between clones, not taking into account the region's length, although this comparison can be made between passages.

**Figure 5:**
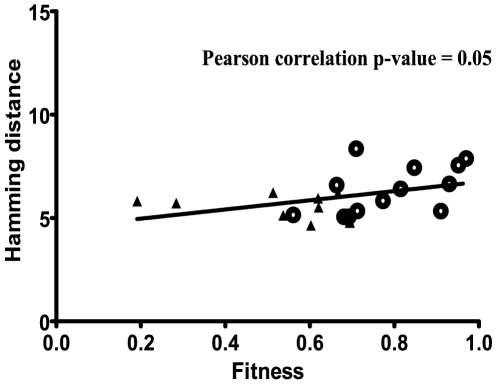
Correlation of heterogeneity and fitness in the HIV-1 clones during the serial passages. Fitness versus quasispecies heterogeneity values per clone are represented. Quasispecies heterogeneity was calculated as total Hamming distance, as explained in Materials and methods. A linear regression analysis between heterogeneity and fitness values was performed. The Pearson correlation test was used to check the values obtained, and the p-value is shown at the top. Symbols are as in Figure 2.

In addition, a global correlation analysis indicated a significant correlation between viral heterogeneity and viral fitness with a p-value = 0.05 (Table 1 and Figure 5). Therefore, fitness increase of HIV-1 can occur without modifications in the consensus sequence of the replicating quasispecies and quasispecies complexity is associated with the increases of HIV-1 fitness.

## Discussion

The present study was designed to investigate the early steps of fitness recovery of ten debilitated HIV-1 clones subjected to only ten serial large population passages. In the course of fitness gain, the genetic variation differed from clone to clone, but a global diversification of viral quasispecies was observed, illustrating that the initial phase of fitness recovery is characterized by the generation of variation. The increase of fitness varied among clones, with higher increases in clones with lower initial values (D1, D1.5 and D2). This fitness augmentation was not related to viral production, as measured by p24 levels, but it was associated with an increase in viral infectivity (Figure 3). Consensus nucleotide sequence of the entire viral genome showed the accumulation of mutations in some passaged populations but not in others. This result documents that fitness alterations can occur without variation of the consensus sequence. Similar results have been previously reported for several other riboviruses [8], [9], [24], [25], [26], [27]. The global fitness correlation with heterogeneity could be differentiated between non-significant correlation in the initial populations and a statistical association in passage 11 viruses (see Figure 5). Fitness increase correlated with quasispecies heterogeneity but not with the number of mutations fixed in the viral populations (Table 1). It must be noted that the experiment was designed to test the initial process of fitness recovery, and that it cannot be excluded that additional passages might result in further fitness increases accompanied by modifications of the consensus sequence. The key point of this cell culture study is that a transient fitness increase can occur without being reflected in the consensus sequence and that critical information in HIV-1 behavior can be lost if no analyses of mutant spectra are performed.

In the case of HIV-1, fitness change without variation in the consensus genomic sequence can be significant in the clinical set-up, because changes in highly relevant phenotypes, for example those associated with antiviral resistance, are generally monitored by the appearance of mutations in the consensus sequence of the viral populations [28]. The role of quasispecies heterogeneity in fitness increase was most evident in the populations that did not display changes in the consensus sequence. However; it may be also operating, at a hidden level of the mutant spectra, as suggested by the comparison of heterogeneity values, in populations that displayed a fitness increase and that incorporated mutations in their consensus sequences.

The experiment reported here was undertaken to simulate population changes likely to occur during HIV-1 natural infections. High HIV-1 population sizes are generally attained at primo-infection, and virus replication is partially controlled by the immune system, primarily by the cytotoxic T cell (CTL) response [29], [30], [31], [32], [33], [34], [35], and later by neutralizing antibodies [36]. Consequently, the viral load drops to a viral set point, which remains stable during the asymptomatic phase, and is variable among patients. Genetic variability studies in HIV-1 patients [37], [38], [39] and in a set of typical progressors have established consistent patterns of viral heterogeneity and divergence from the initial transmission to AIDS [40].

The present study with HIV-1 reinforces the adequacy of quasispecies theory to interpret retrovirus evolution. The key observation is that fitness recovery cannot only be mediated by the fixation and dominance of advantageous mutations but also by an increase in quasispecies heterogeneity. Thus, the viral population as a whole is the one involved in the fitness recovery. This result supports the concept that the target of selection is not a single genome but the complete quasispecies [5], [41], [42]. Dominance of specific mutants is not a requirement for fitness gain, and internal interactions within quasispecies can affect retroviral evolution.

## Materials and Methods

### Cells, viruses and biological cloning

The HIV-1 parental population was isolate s61 [2], obtained by standard co-culture procedures, from a 4-year-old child with AIDS symptoms [2]. From this viral population, six biological clones D, G, E, H, I, and K were derived by plating on MT-4 cells [43]. These clones were subjected to serial plaque-to-plaque transfers to obtain populations D15, E11, G7, H12, I15 and K15 [3] (Figure 1). From these viral populations, two clones were obtained from lineages D, G, K, and I, and only one clone could be recovered from lineages E and H (Figure 1).

### Large population passages of viruses

Clones D1, D2, E1, G1, G2, H1, I1, I5, K1, and K2 were subjected to ten large population passages by infecting 5×10^6^ or 2.5×10^6^ MT-4 cells at a multiplicity of infection (M.O.I.) of about 0.01 TCID_50_/cell (Figure 1). Viral titers were obtained in MT-2 cells and are expressed as the 50% tissue culture infective dose per ml (TCID_50_/ml). Virus was recovered from the culture supernatant when cytopathology was complete (5 to 7 days post-infection). For each subsequent passage, fresh MT-4 cells were infected with the same volume of the supernatant from the previous passage, taking into account that viral titers do not change significantly during serial passages in MT-4 cells [2]. p24 determination was carried out in the culture supernatant with an automated enzymatic method in an Elecsys 2010 analyser using a p24 detection kit (Roche Diagnostics, HIV Ag.HIV (groups M and 0) p24 antigen, ref.11971611 122). Cells were cultured in closed bottles and all the procedures were aimed at minimizing the possibility of cross-contamination, which was monitored with uninfected control cultures maintained in parallel. No evidence of contamination was observed at any time.

### Fitness assay

Relative fitness values were determined by growth-competition experiments as previously described [44]. Briefly, the assay consists in the coinfection of MT-4 cells with a known number of TCID_50_/ml of the virus to be tested and of a reference clone (J1). The genome of clone J1 contains a deletion of 45 nucleotides in the region of *env* gene that encodes the V1-V2 loop; this deletion is readily detectable in a GeneScan analysis. Coinfections, performed in duplicate or triplicate, were carried out for a maximum of 5 passages. The proportion of the two viruses in each passage was quantified by Gene Scan analysis. The fitness values per competitive transfer series were calculated from the slope of exponential plot as previously described [44]. The statistical significance of fitness differences between the initial and final populations was tested using the Graphpad software.

### RNA extraction and PCR amplification for complete genome analysis

Consensus genomic nucleotide sequences for the entire HIV-1 genome were determined for the initial and final viral populations as previously described [4]. Sequences were derived from viral RNA in the supernatant of the cell cultures. RNA was extracted with a guanidinium isothiocyanate lysis buffer and glass milk as previously described [4]. RT-PCRs used for the determination of the consensus genome sequence were performed using at least 2,000 copies of viral RNA as starting template. The first amplification was carried out using the Access RT-PCR System (Promega) with 6 different pairs of oligonucleotide primers that cover the entire HIV-1 genome. Internal amplifications were performed with the EcoTaq DNA polymerase (Ecogen). The sequence of the primers used in the amplifications, covering the 5′LTR-gag (1880 bp), pol-vpr (3450 bp) and vpr-3′LTR (3923 bp) regions, will be made available upon request. Both external and internal amplifications involved 35 cycles with temperatures chosen according to the composition of the primers. Negative amplification controls were run in parallel to monitor absence of contamination. Primer location and numbering of nucleotide changes are referred to isolate HIV-1 HXB2 included in the Los Alamos data base [45]. Sequence was determined on the two cDNA strands with an ABI 373 automatic sequencer. Multiple sequence alignments were performed using the CLUSTAL W program [46]. Sequences have been deposited in GeneBank with the accesion numbers GQ386774 to 386794.

### GeneScan Analysis

For fitness determination, to avoid recombination during the reverse transcriptase reaction, quantification of the viruses was performed on proviral DNA. Prior to this decision, a comparison between estimations performed on proviral DNA or from viral RNA from the culture supernatant showed no statistical differences (data not shown). DNA was extracted from the cellular pellets of the competition cultures by standard phenol-chlorophorm method. The region encoding the V1/V2 loops of gp120 of the competing viruses, which were different in length, was amplified by PCR using one of the primers labelled with the fluorescent dye 6-FAM. The primers used were: 34, 5′-6FAM-GTCACAGTCTATTATGGGGTACCTGTGT-3′; and 31, 5′-ACCTCAGTCATTACACAGGCCTGCAGCGC-3′. The PCR products were diluted from 2-to 10-fold with water to obtain peak intensities in the linear range, and 1 µl of these dilutions were mixed with 9 µl of ROX 1000 size standard (Applied Biosystems) previously diluted 17-fold with formamide. Samples were run on an ABI PRISM 3700 machine, and data were analysed using GeneMapper v 4.0. Peak areas were used to calculate the fitness of each virus versus the standard J1.

### Quasispecies analysis and calculation of population heterogeneity

For the analysis of mutant spectra we selected four genomic regions that were amplified using three primers pairs. The first pair amplified RNA positions 672 to 1363 of HXB2 (692 nucleotides) that spanned the leader sequence and the p17 protein in the *gag* gene. The second pair amplified RNA positions 6045 to 6734 (711 nucleotides) emcompassing *vpu* and the V1–V2 region of *env*. The third pair amplified RNA positions 7039 to 7641 (602 nucleotides) that comprised the V3–V4 region of *env*. In total, the three amplifications covered four regions: the 5′ LTR/p17, *vpu-*, *env* (V1–V2)- and *env* (V3–V4)-coding regions which represent a total of −2,005 genomic residues. At least 20 independent clones were used to represent the viral quasispecies of each population at each passage. The RT-PCRs products were cloned using TA TOPO cloning (Invitrogen), following the manufacturer's instructions. In each region, average Hamming distance was calculated for every virus using the MEGA v4.0 software. A sum of the average Hamming distances in the 2,005 nucleotides analyzed was used for the analysis showed in Figure 4 and 5.

### Statistical analysis of the results

Wilcoxon signed rank tests were applied to compare the different parameters used to characterize the viral populations. The p-values obtained are shown in each Figure, and the sum of signed ranks are indicated in the corresponding legends. A Pearson test of correlation was used to study the possible association between viral heterogeneity and fitness.
